# Electroacupuncture Ameliorates Learning and Memory Deficits in Vascular Cognitive Impairment Rats Through Activation of the Supramammillary Nucleus–Dentate Gyrus Circuit

**DOI:** 10.1002/cns.70962

**Published:** 2026-06-12

**Authors:** Fang Wan, Lu Wang, Qing‐Yong Wang, Jing‐Wen Yang, Cun‐Zhi Liu

**Affiliations:** ^1^ International Acupuncture and Moxibustion Innovation Institute, School of Acupuncture‐Moxibustion and Tuina Beijing University of Chinese Medicine Beijing China

**Keywords:** dentate gyrus, electroacupuncture, supramammillary nucleus, synaptic plasticity, vascular cognitive impairment

## Abstract

**Aims:**

This study aimed to elucidate the potential mechanism, especially neural circuit mechanisms, through which electroacupuncture (EA) mitigates cognitive decline in a rat model of vascular cognitive impairment (VCI).

**Methods:**

Using a rat two‐vessel occlusion (2VO) model, we assessed the therapeutic impact of EA on spatial and non‐spatial memory. We integrated in vivo fiber photometry and immunofluorescence to monitor neuronal activities in the dentate gyrus (DG) and supramammillary nucleus (SuM), while Golgi staining was employed to evaluate hippocampal synaptic plasticity. Viral tracing and chemogenetic manipulations were utilized to characterize the functional necessity of the SuM^Glu^‐DG circuit.

**Results:**

EA at ST36 significantly restored learning and memory function. This functional recovery was accompanied by improved synaptic plasticity and heightened neuronal activity in the DG. Fiber photometry revealed that EA specifically activated glutamatergic neurons in the SuM, a key upstream regulator of the DG. Notably, targeted chemogenetic inhibition of the SuM^Glu^‐DG circuit abolished the cognitive improvements typically induced by EA.

**Conclusion:**

Our findings demonstrate that EA ameliorates learning and memory deficits in VCI through regulating the SuM^Glu^‐DG circuit.

## Graphical abstract

Electroacupuncture at ST36 alleviates cognitive deficits and restores hippocampal synaptic plasticity in VCI rats by activating the SuM^Glu^‐DG circuit. This study identifies the SuM^Glu^‐DG circuit as an essential mediator for the therapeutic and neuroprotective effects of acupuncture in vascular‐induced cognitive decline.

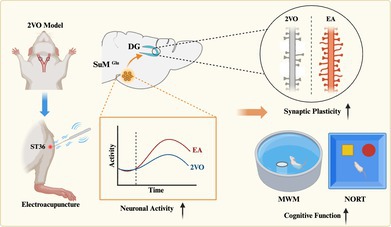

## 1 Introduction

Against the backdrop of a rapidly expanding aging population, vascular cognitive impairment (VCI) has emerged as a major public health challenge on a global scale. VCI development is primarily driven by cerebrovascular impairment, specifically protracted cerebral hypoperfusion resulting from macrovascular pathologies or microangiopathy [1]. Persistent hypoperfusion triggers a detrimental cascade that eventuates in synaptic failure and neuronal attrition, consequently precipitating lesions in brain regions like the hippocampus, cortex, cerebellum, and basal ganglia [2, 3]. Each year, approximately 10 million new cases of cognitive impairment are reported, with roughly half being either of or attributed to vascular origins [4]. To date, no specific treatment for VCI is currently available, and management of risk factors such as hypertension and diabetes is recommended. Drugs commonly prescribed for Alzheimer's dementia (AD), including memantine and cholinesterase inhibitors, lack robust and consistent evidence of efficacy in the treatment of patients with VCI [5].

Synaptic plasticity stands as the core neurobiological substrate of cognition, and impairment thereof is identified as an early pathological event in VCI [6]. As a brain region that plays a critical role in cognitive function, the hippocampus is particularly vulnerable to ischemic injury. Accumulating evidence has confirmed that the hippocampus in VCI mice undergoes substantial neuronal loss and synaptic damage [7]. As a hippocampal subregion, the DG is considered the “gateway” to the hippocampus and is essential for the orchestration of spatial memory formation [8]. Notably, marked volumetric atrophy of the DG has been documented in patients with cerebrovascular disease [9]. Beyond localized damage, emerging evidence has turned the focus toward upstream modulatory inputs, specifically the supramammillary nucleus (SuM) [10, 11]. As a functionally pleiotropic hypothalamic subregion, the SuM governs core cognitive processes, including memory encoding and reinforcement learning. Glutamatergic neurons within the SuM (SuM^Glu^) send robust projections to the DG, thereby regulating hippocampal neuronal activity. Current research indicates that this SuM‐driven “novelty signal” is essential for modulating hippocampal memory encoding. Furthermore, optogenetic activation of the SuM^Glu^–DG circuit has been shown to modulate contextual memory consolidation, corroborating the circuit's status as an indispensable node in the neural network subserving cognitive processes [12].

Acupuncture is recognized for its broad clinical utility in managing various cerebrovascular disorders. In the context of VCI, growing evidence indicates that acupuncture improves cognitive function and other clinical outcomes in VCI patients [13, 14, 15]. Electroacupuncture (EA) at ST36 (Zusanli) is particularly notable; it has been shown to exert neuroprotective effects by modulating inflammatory pathways and oxidative stress [16, 17]. While our previous research demonstrated that EA at ST36 effectively rescues hippocampal synaptic plasticity in VCI models [18], the mechanisms that orchestrate these improvements remain elusive. Specifically, whether the SuM contributes to this EA‐induced synaptic recovery, as well as the specific contribution of the SuM‐DG circuit in mediating EA's beneficial effects, remains unclear.

In this study, we demonstrated that in the two‐vessel occlusion (2VO) rat model, EA enhanced synaptic plasticity and neuronal activity in the DG, thereby improving learning and memory deficits. By employing a combination of fiber photometry recordings, viral tracing, and chemogenetic manipulations, we also validated that the SuM^Glu^‐DG circuit is crucially involved in the therapeutic effects of EA.

## 2 Methods

### 2.1 Animals

The study was conducted using 8 week‐old male Wistar rats, which were maintained at the Laboratory Animal Center of Beijing University of Chinese Medicine. Animals were housed in a temperature‐controlled facility (23°C–25°C) with a standardized 12 h light/dark cycle and provided with ad libitum access to both food and water. To ensure physiological stability, a minimum 1 week period of acclimatization was mandatory prior to the commencement of any experimental procedures. All experimental protocols were reviewed and formally approved by the Institutional Animal Care and Use Committee of Beijing University of Chinese Medicine (Permit Number: BUCM‐4‐2,020,091,601‐3063).

### 2.2 Experimental Design and Animal Allocation

To evaluate the therapeutic effects of EA, we performed behavioral testing, Golgi staining, and immunofluorescence staining. The rats were randomly assigned to 5 groups: Sham, Sham + EA, 2VO, 2VO + EA, and 2VO + Sham EA (SEA). Within each group, independent subsets of animals were utilized to ensure that different testing procedures did not interfere with one another. Specifically, one subset (*n* = 6 per group) was subjected to behavioral tests; a second subset (*n* = 9 per group) was dedicated to Golgi staining; and a third subset (*n* = 3 per group) was utilized to immunofluorescence staining.

To investigate the effects of EA on neuronal activity in the DG and SuM, we performed in vivo fiber photometry using two independent animal cohorts, each targeting one specific region. A longitudinal within‐subject design (*n* = 6 per group for each cohort) was employed to record calcium signals from the same animals across five sequential time points: Pre‐2VO, Post‐2VO, and EA days 1, 7, and 14.

To evaluate the specific necessity of the SuM^Glu^‐DG circuit in mediating the effects of EA, we first verified the structural connectivity between the SuM and DG using viral tracing. Following this verification, we inhibited the SuM^Glu^‐DG circuit in 2VO rats using chemogenetic manipulations. The rats were randomly assigned to 4 groups: 2VO + mCherry + CNO, 2VO + hM4D(Gi) + CNO, 2VO + hM4D(Gi) + Saline + EA, and 2VO + hM4D(Gi) + CNO + EA (*n* = 6 per group).

### 2.3 2VO Surgery

The method for preparing 2VO rats has been described in our previous study [18]. Rats were induced with 3% isoflurane and maintained on a heating platform to ensure a stable core temperature of 37°C. After dissecting the subcutaneous tissue, the bilateral common carotid arteries were meticulously isolated from the vagus nerves and occluded by permanent 5–0 silk ligation (Jinhuan, 5–0, China). 2% ampicillin (0.25 mL/100 g) was delivered via intraperitoneal injection to prevent postoperative infection. Post‐surgical wounds were sutured, and animals were monitored until full anesthetic recovery before rehousing. The sham group underwent identical surgical manipulations, excluding the arterial ligation step.

### 2.4 EA Intervention

EA intervention was initiated on the third postoperative day following 2VO induction. Stainless‐steel acupuncture needles (0.16 × 13 mm, Beijing Zhongyan Taihe Medical Instrument Co. Ltd., Beijing, China) were inserted at the bilateral ST36 acupoints, situated 5 mm below the fibular head and 2 mm lateral to the tibial anterior tubercle. Needle insertion reached a depth of 5–7 mm, with electrical stimulation delivered via a HANS‐200A device (1 mA intensity; 2/15 Hz frequency) for 20 min. This specific parameter was selected to reduce EA adaptation, which is consistent with our previous work [19].

The 2VO + SEA groups received shallow needle insertion (depth: 1–2 mm) at the bilateral hypochondrium, 10 mm superior to the iliac crest, without electrical stimulation. The Sham groups were subjected to identical restraint procedures in the absence of needle application. All groups received daily treatment for 2 weeks, with 1 day of rest after every 6 consecutive treatments.

### 2.5 Behavioral Tests

Spatial acquisition and memory retention were assessed utilizing the Morris water maze (MWM) following established protocols [20]. Prior to formal assessment, a visible platform trial was conducted to identify and exclude animals with visual or motor impairments. During the 5 day acquisition phase, a circular escape platform was submerged in the center of the third quadrant. Rats were introduced into the pool from randomized starting locations daily, with a 90 s maximum duration allotted to locate the platform. Successful animals remained on the platform for 20 s; conversely, those failing to find the target within the time limit were manually guided to it for a 20 s rest, with their escape latency recorded as 90 s. On day 6, a 90 s probe trial was performed by removing the hidden platform to evaluate memory retention. Key metrics, including swimming velocity, time expended in the target quadrant, and number of platform crossings, were recorded and analyzed.

Novel object recognition test (NORT) was performed to assess non‐spatial memory as described previously [21]. The behavioral trials were performed in a black rectangular chamber measuring 100 × 100 × 40 cm. During the initial acquisition phase, rats were permitted 10 min of spontaneous interaction with two identical objects. Following a 6 h retention interval, animals returned to the arena where one familiar object was replaced by a novel stimulus for a further 10 min exploration period. To quantify cognitive performance, the discrimination index was determined by dividing the duration of novel object investigation by the total exploration time for both items.

Open field test (OFT) was performed to evaluate the locomotor activity and anxiety‐like behaviors, as previously described [22]. The experimental enclosure was a black plastic arena measuring 100 × 100 × 40 cm, featuring a demarcated central zone on the base. Each subject was placed in the center and allowed 10 min of autonomous exploration. To prevent olfactory interference, the apparatus was thoroughly sanitized with an ethanol solution following each session. An overhead camera system recorded the cumulative distance traveled and the duration of occupancy within the center region.

### 2.6 Golgi Staining

To visualize dendritic morphology, Golgi‐Cox staining was executed utilizing a commercial kit (PK401, FD NeuroTechnologies) in strict adherence to the manufacturer's protocol. Rats were anesthetized intraperitoneally with 1% pentobarbital sodium. Whole brains were harvested and immediately submerged in a mixture of Solutions A and B, which were then stored in a dark environment at ambient temperature. After 2 weeks, the tissues were transferred to Solution C and incubated for 1 week. Subsequently, the brain samples were processed into 100‐μm‐thick coronal slices. The sections were placed in a 6‐well plate filled with double‐distilled water and washed twice. After staining with Solution D + E and dehydration through a graded ethanol series, the sections were cleared in xylene and cover slipped with neutral balsam mounting medium. Photomicrographs were captured via an Olympus system (VS‐120, Japan), and dendritic parameters were quantified using ImageJ software (1.52a, National Institutes of Health, USA). Specifically, dendritic spine density was quantified on the tertiary dendrites, with 3–5 well‐impregnated neurons randomly selected and analyzed per rat.

### 2.7 Immunofluorescence Staining

Following decapitation, rat brain tissues were harvested and processed into cryosections. After three PBST rinses, the slices underwent a 1 h blockade at 37°C in a buffer composed of 0.7% Triton X‐100 and 5% donkey serum. Subsequently, sections were exposed to primary antibodies targeting c‐Fos (rabbit anti‐c‐Fos, 1:1000, Cell Signaling Technology) and CaMKIIα (mouse anti‐CaMKIIα, 1:200, Proteintech) for 48 h at 4°C. Following additional triple washes, the tissue was incubated with secondary antibodies—specifically, DyLight 594‐conjugated Goat anti‐rabbit IgG (1:1000, Abcam) and DyLight 488‐conjugated Goat anti‐mouse IgG (1:1000, Abcam)—for 1 h at 37°C. The sections were then washed three times, mounted with DAPI‐containing Fluoroshield, and sealed with coverslips. Fluorescence visualization was performed using an Olympus VS‐120 virtual slide system. For data analysis, the number of c‐Fos and CaMKIIα co‐labeled neurons was manually quantified using ImageJ software (v1.52a) by an investigator blinded to the experimental groupings.

### 2.8 Fiber Photometry Recording

The rAAV‐hSyn‐GCaMP6s was injected into the DG (coordinates AP = −3.72 mm; ML = 2.2 mm; DV = −3.2 mm), and rAAV‐CaMKIIα‐GCaMP6s was injected into the SuM (coordinates AP = −4.5 mm; ML = 0 mm; DV = −8.9 mm), respectively. All stereotaxic coordinates were determined relative to Bregma, according to The Rat Brain in Stereotaxic Coordinates (Paxinos and Watson, 2006 [23]). An optic fiber (ID: 200 μm, OD: 2.5 mm, FiberMD 200 μm, NA: 0.37, Thinker Tech, China) was implanted 0.2 mm above the injection site in the DG or SuM. After 3 weeks of viral expression, fiber photometry recordings were performed using a commercial device (QAXK‐FPS‐03, Thinker Tech, China). In brief, the 470 nm and 410 nm laser beams were first launched into the fluorescence cube and then into the optic fibers. The 410 nm laser was used for motion artifact correction. GCaMP6s‐derived and control emission fluorescence were collected by the camera at 40 Hz. In vivo recordings were performed before and after 2VO surgery, as well as post‐EA days 1, 7, and 14, with each recording session lasting 20 min. All recordings were conducted while the rats were under light anesthesia (isoflurane maintained at 1%). We derived the photometry signal F as the ratio F470/F405, and calculated ∆F/F = (F—F0)/F0, where F0 is the median of F. The average of the peak ∆F/F values and the area under the curve (AUC) were quantified and analyzed for each rat.

### 2.9 Viral Tracing

To delineate the neuroanatomical connectivity between the SuM and the DG, viral tracing was performed. Under 3% isoflurane anesthesia, rats were secured within a stereotaxic apparatus (RWD Life Science, Shenzhen, China). For retrograde labeling, rAAV‐Retro‐CaMKIIα‐EGFP (300 nL) was microinjected into the DG (coordinates were positioned relative to Bregma: AP −3.72 mm, ML ±2.2 mm, DV −3.3 mm) at a constant infusion rate of 0.3 nL/s. The rAAV‐CaMKIIα‐mCherry (300 nL) was delivered into the SuM (coordinates were positioned relative to Bregma: AP −4.5 mm, ML 0 mm, DV −8.9 mm) using identical flow parameters to facilitate anterograde mapping. Following a 21 day period to ensure robust viral expression, brain tissues were harvested and processed for cryosections.

### 2.10 Chemogenetic Manipulation

To investigate whether the SuM^Glu^‐DG circuit is essential for the therapeutic efficacy of EA, a chemogenetic approach was employed. Specifically, rAAV‐Retro‐CaMKIIα‐Cre (300 nL) was microinjected into the DG, while rAAV‐EF1α‐DIO‐hM4D(Gi)‐mCherry (300 nL) or a control rAAV‐EF1α‐DIO‐mCherry vector was delivered into the SuM. All intracranial infusions were maintained at a constant rate of 0.3 nL/s. 3 weeks following viral transduction, rats underwent 2VO surgery, with therapeutic interventions commencing after a 3 day postoperative recovery interval. Selective circuit inhibition was achieved through the intraperitoneal administration of clozapine N‐oxide (CNO; 1 mg/kg; BrainVTA, China). CNO was administered 30 min prior to each daily EA intervention, with an administration frequency matching the EA cycle. All viral constructs were synthesized by BrainCase (China) unless otherwise indicated.

### 2.11 Statistical Analyses

All statistical evaluations were executed using IBM SPSS Statistics (v22.0), while GraphPad Prism (v9.5) was utilized for data visualization. Quantitative results are expressed as the mean ± Standard Error of the Mean (𝜒¯ ± SEM). Distribution normality was verified for all datasets; for non‐normally distributed data, the Kruskal‐Wallis test was implemented. In instances where data satisfied both normality and variance homogeneity (assessed via Levene's test), one‐way analysis of variance (ANOVA) was performed, followed by Tukey's post hoc analysis. If the assumption of equal variance was violated, multiple comparisons were conducted using Dunnett's T3 test. For pairwise comparisons, if normality was not met, the Wilcoxon Signed‐Rank test was employed. If the data met the criteria for normality, the Paired Samples *t*‐test was employed. Additionally, two‐way repeated‐measures ANOVA was employed to assess group differences in data across multiple time points in the MWM test. All statistical tests were considered statistically significant at *p < 0.05*.

## 3 Results

### 3.1 EA Alleviated Learning and Memory Deficits in VCI Rats

**FIGURE 1:**
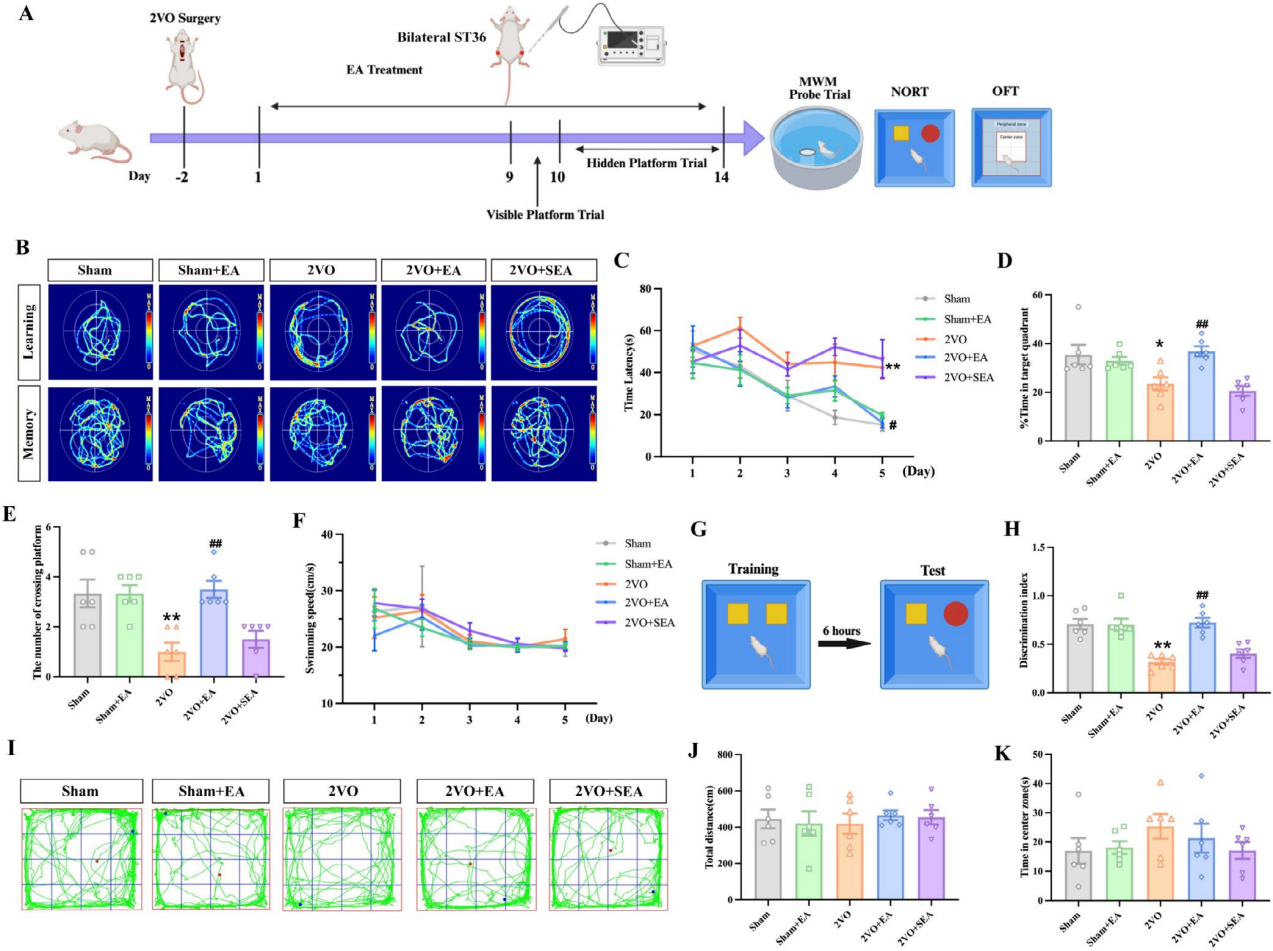
EA improved cognitive impairment in VCI rats. (A) Timeline of the experimental procedure. Abbreviations: 2VO, two‐vessel occlusion; EA, electroacupuncture; MWM, Morris water maze; NORT, novel object recognition test; OFT, open field test. (B) Representative swimming trajectories throughout the MWM test. (C) Average latencies for locating the submerged platform during the spatial navigation phase (learning) (Two‐way ANOVA, *n* = 6 rats per group, Time: F_4,100_ = 11.784, *P < 0.001*; Groups: F_4,25_ = 11.019, *P < 0.001*; Group*Time: F_16,100_ = 1.326, *P > 0.05*; Day 5: One‐way ANOVA, *n* = 6 rats per group, F_4,25_ = 9.454, * P < 0.001*, post hoc Dunnett's test: ** P < 0.01 vs. Sham group, ^#^ *P < 0.05*vs 2VO group). (D) Percentage of swimming time in the target quadrant during the probe test (memory) (Kruskal–Wallis test, *n* = 6 rats per group, *P < 0.05 vs. Sham group, ^##^ *P < 0.01* vs. 2VO group). (E) The number of platform crossings in the probe test (memory) (Kruskal–Wallis test, *n* = 6 rats per group, **P < 0.01 vs. Sham group, ^##^ *P < 0.01* vs. 2VO group). (F) Average swimming speed in the MWM test (Two‐way ANOVA, *n* = 6 rats per group, Time: F_4,100_ = 8.960, *P < 0.001*; Groups: F_4,25_ = 0.430, *P > 0.05*; Group*Time: F_16,100_ = 0.345, *P > 0.05*; Day 5: One‐way ANOVA, *n* = 6 rats per group, F_4,25_ = 0.429, *P > 0.05*). (G) The schematic for the NORT. (H) The discrimination index in the NORT (Kruskal–Wallis test, *n* = 6 rats per group, **P < 0.01 vs. Sham group, ^##^ *P < 0.01* vs. 2VO group). (I) Representative traces in the OFT. (J) Total traveled distance in the OFT (One‐way ANOVA, *n* = 6 rats per group, F_4,25_ = 0.167, *P > 0.05*). (K) Time in the center area in the OFT (One‐way ANOVA, *n* = 6 rats per group, F_4,25_ = 0.855, *P > 0.05*).

To investigate the effects of EA on cognitive functions in VCI rats, the MWM test and NORT were performed (Figure 1A). In the MWM place navigation test, repeated‐measures ANOVA demonstrated that the escape latency of each group decreased significantly with the increase in the number of training days. As shown in Figure 1B,C 2VO rats exhibited significantly prolonged escape latency compared to the Sham group, indicating impaired spatial acquisition. However, EA treatment effectively mitigated these learning deficits, as evidenced by a progressive reduction in the time taken to locate the platform. No significant difference was observed between the 2VO and 2VO + SEA groups. Spatial memory retention (Figure 1D,E) was further evaluated during the probe trial. Consistent with the learning phase, 2VO rats spent significantly less time in the target quadrant and showed fewer platform crossings compared with the Sham group. These memory impairments were reversed by EA intervention. Average swimming speed remained consistent across all experimental groups (Figure 1F), indicating that the observed disparities in learning and memory were not confounded by motor capability.

Regarding the NORT (Figure 1G,H), results revealed that 2VO induction led to a significant reduction in discrimination indices compared to Sham animals, suggesting impaired non‐spatial memory. However, compared with the 2VO group, the 2VO + EA group exhibited significantly improved non‐spatial memory, as evidenced by a higher discrimination index.

The OFT was performed to evaluate anxiety in VCI rats. As shown in Figure 1I–K, the results demonstrated that cumulative ambulatory distance and duration within the central arena remained statistically indistinguishable across all experimental groups, indicating that the motor activity and anxiety‐like behavior of rats were not affected by 2VO or EA treatment.

### 3.2 EA Improved Synaptic Plasticity and Neuronal Activity in the DG

**FIGURE 2:**
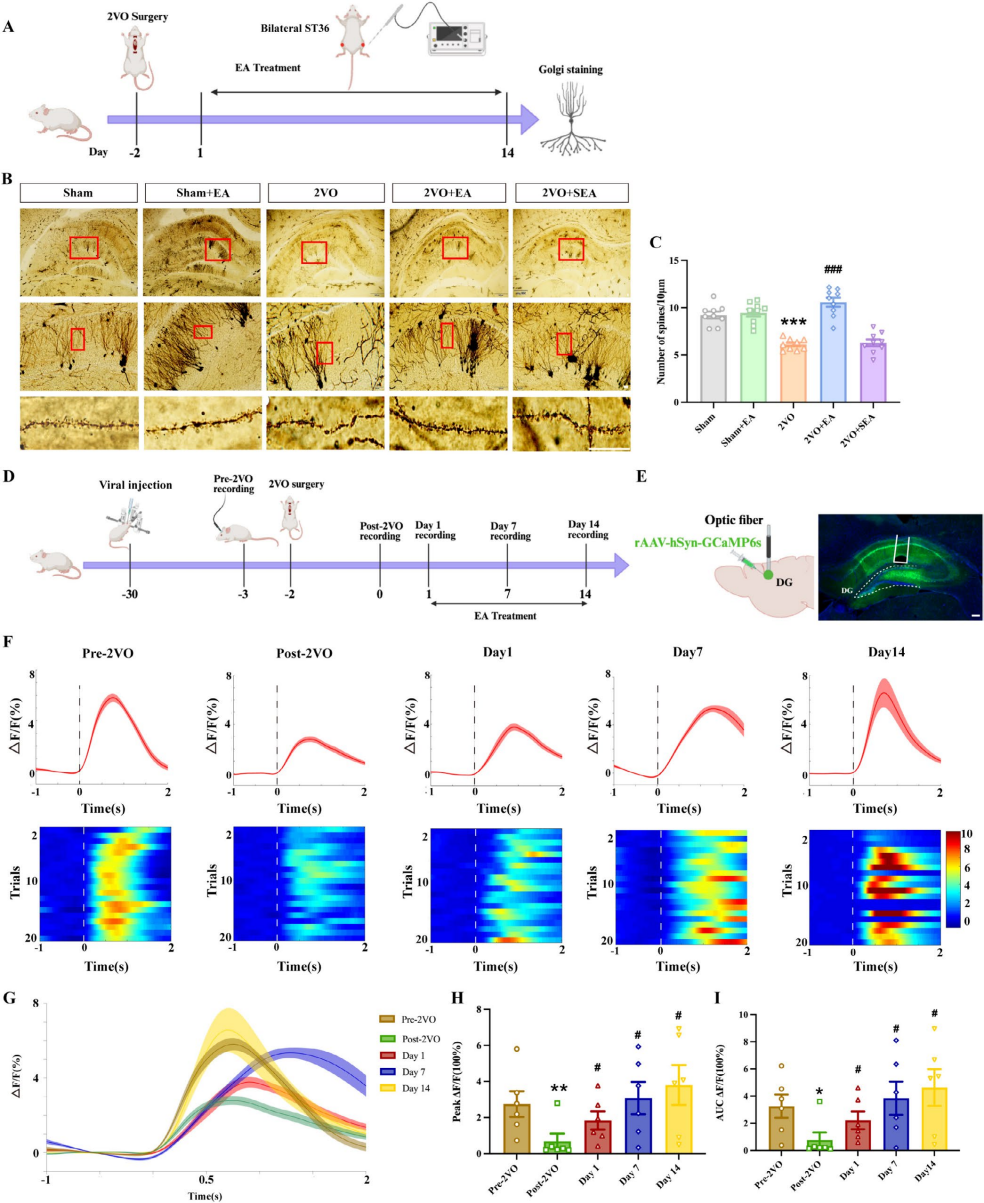
EA improved synaptic plasticity and neuronal activity of DG in VCI rats. (A) Timeline of the Golgi staining. (B) Representative images of Golgi staining in the DG. Scale bar: 20 μm. (C) Quantitative analysis of dendritic spine density in DG (One‐way ANOVA, *n* = 9 rats per group, F_4,40_ = 30.312, *P < 0.001*, post hoc Tukey's test: ***P < 0.001 vs. Sham group, ^###^ *P < 0.001* vs. 2VO group). (D) Timeline of the fiber photometry recording. (E) Schematic of the optical fiber photometry assay and representative images of EGFP expression in the DG. Scale bar: 200 μm. (F) Heatmaps and average of calcium activities of DG neurons. (G) The changes in calcium activities of neurons in the DG at different time points. (H) Peak of Ca^2+^ activity in DG neurons at different time points (Paired Samples t‐test, *n* = 6 rats per group, **P < 0.01 vs. Pre‐2VO, ^#^ *P < 0.05* vs. Post‐2VO). (I) Area under the curve of Ca^2+^ activity in DG neurons at different time points (Paired Samples t‐test, *n* = 6 rats per group, *P < 0.05 vs. Pre‐2VO, ^#^ *P < 0.05* vs. Post‐2VO).

Dendritic spines are the fundamental structural units of synaptic plasticity. To explore the effects of EA on synaptic plasticity in VCI rats, we assessed the density and morphology of dendritic spines using Golgi staining (Figure 2A). As shown in Figure 2B,C, the 2VO group exhibited a significantly decreased dendritic spine density compared with the Sham group, indicating impaired integrity of dendritic spines. EA treatment significantly ameliorated synaptic damage, as evidenced by increased dendritic spine density compared with the 2VO group. No significant difference was observed between the 2VO group and the 2VO + SEA group.

To characterize the impact of EA on DG neuronal activity, we injected rAAV‐hSyn‐GCaMP6s into the DG and recorded calcium signals before and after 2VO surgery, as well as on the 1st, 7th, and 14th days post‐EA, via fiber photometry (Figure 2D). Post‐experimental histological verification confirmed targeted GCaMP6s expression and the precise anatomical positioning of the implanted fiber (Figure 2E). As shown in Figure 2F–I, compared to the baseline (Pre‐2VO), the calcium signaling in DG neurons of rats significantly decreased after 2VO surgery (Post‐2VO), which was evidenced by decreased area under the curve (AUC) and mean peak amplitude. However, compared to the Post‐2VO, EA intervention at 1, 7, and 14 days significantly increased the AUC and mean peak amplitude of DG in a time‐dependent manner.

### 3.3 EA Activated Glutamatergic Neurons in the SuM of VCI Rats

**FIGURE 3:**
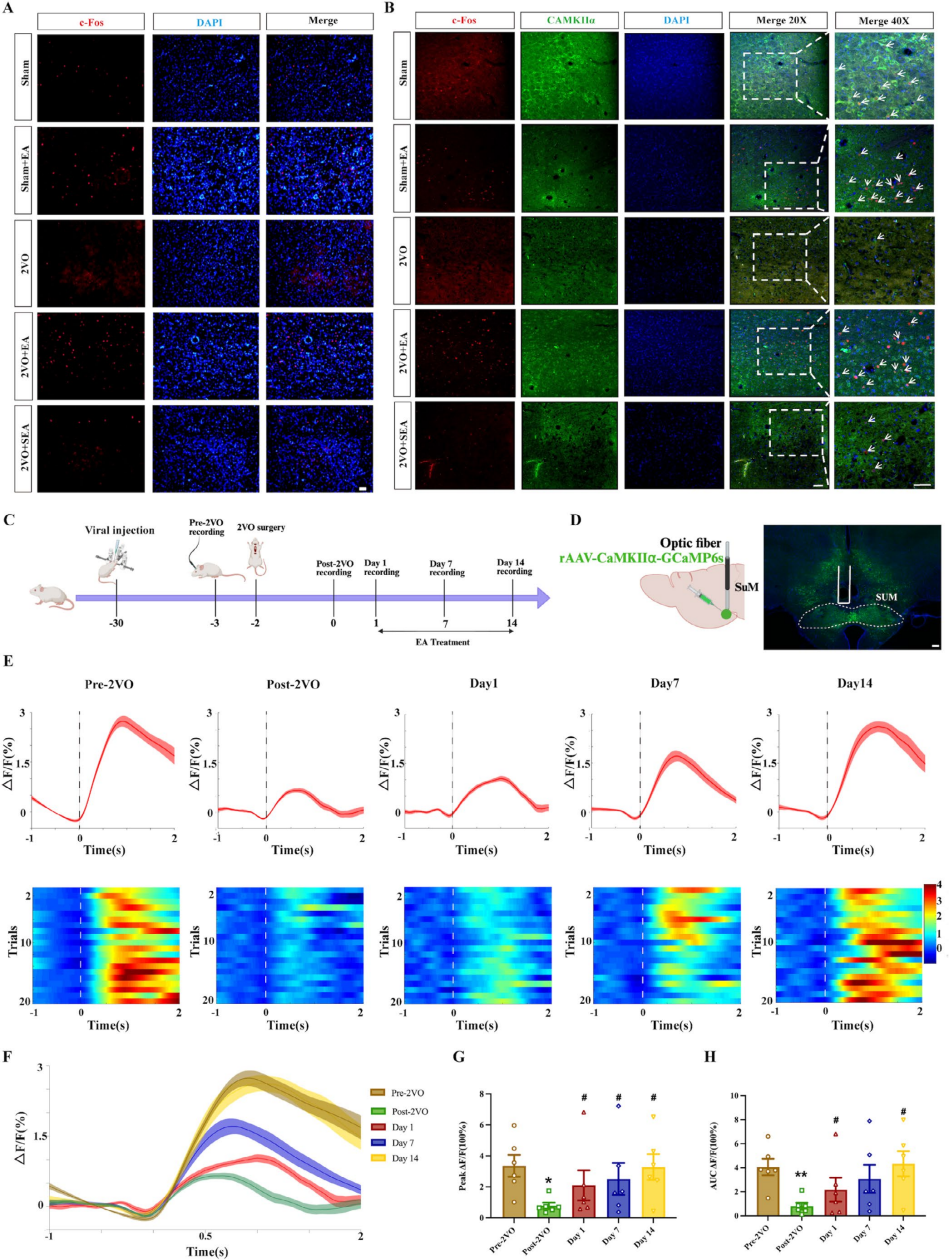
EA enhanced glutamatergic neuronal activity in the SuM. (A) Representative images of c‐Fos (red) expression in SuM. Scale bar: 200 μm. (B) Confocal imagery illustrating the co‐expression of c‐Fos (red) and CaMKIIα (green) neurons. White arrows denote triple‐positive signals (c‐Fos, CAMKIIα and DAPI). Scale bar: 50 μm. (C) Schedule of the fiber photometry recording. (D) Diagrammatic representation of the in vivo photometry configuration alongside characteristic fluorescence images of EGFP‐expressing neurons within the SuM. Scale bar: 200 μm. (E) Heatmaps and average of calcium activities of SuM^Glu^. (F) The changes in calcium activities of SuM^Glu^ at different time points. (G) Peak of Ca^2+^ activity in SuM^Glu^ at different time points (Wilcoxon Signed‐Rank test, *n* = 6 rats per group, *P < 0.05 vs. Pre‐2VO, ^#^ *P < 0.05* vs. Post‐2VO). (H) Area under the curve of Ca^2+^ activity in SuM^Glu^ at different time points (Wilcoxon Signed‐Rank test, *n* = 6 rats per group, **P < 0.01 vs. Pre‐2VO, ^#^ *P < 0.05* vs. Post‐2VO).

Given that the SuM is a vital modulator of hippocampal activity [12, 24], we investigated whether the SuM is involved in the neuroprotective effects of EA. We first examined the expression of c‐Fos, a marker of neuronal activation, within the SuM. As shown in Figure 3A, the neuronal activity was significantly suppressed in the 2VO group as indicated by a marked reduction in c‐Fos‐positive cells. Remarkably, EA treatment effectively activated the neurons in the SuM, significantly increasing the number of c‐Fos‐positive neurons. The SuM is comprised mostly of glutamatergic neurons and, to a lesser extent, of GABAergic and dopaminergic neurons. Given that SuM^Glu^ densely innervate the DG, we next determined whether the EA‐activated neurons were glutamatergic neurons. Double immunofluorescence staining for c‐Fos and the neuronal marker CaMKIIα was conducted, and most c‐Fos‐positive neurons were co‐labeled with CaMKIIα positive neurons (Figure 3B).

To further characterize the functional involvement of the SuM^Glu^ neurons in the effects of EA, in vivo changes in Ca^2+^ signaling were recorded via fiber photometry. The rAAV‐CaMKIIα‐GCaMP6s was injected into the SuM to specifically label glutamatergic neurons (Figure 3C,D). As shown in Figure 3E–H, similar to our observations in the DG, 2VO induction led to a significant collapse of Ca^2+^ activity in SuM^Glu^ neurons. EA intervention triggered the recovery of these calcium signals. This restorative effect followed a significant time‐course, with AUC and mean peak amplitudes values progressively increasing from day 1 to day 14 of treatment.

### 3.4 The SuM^Glu^ ‐DG Circuit Mediated the EA Therapeutic Effects

**FIGURE 4:**
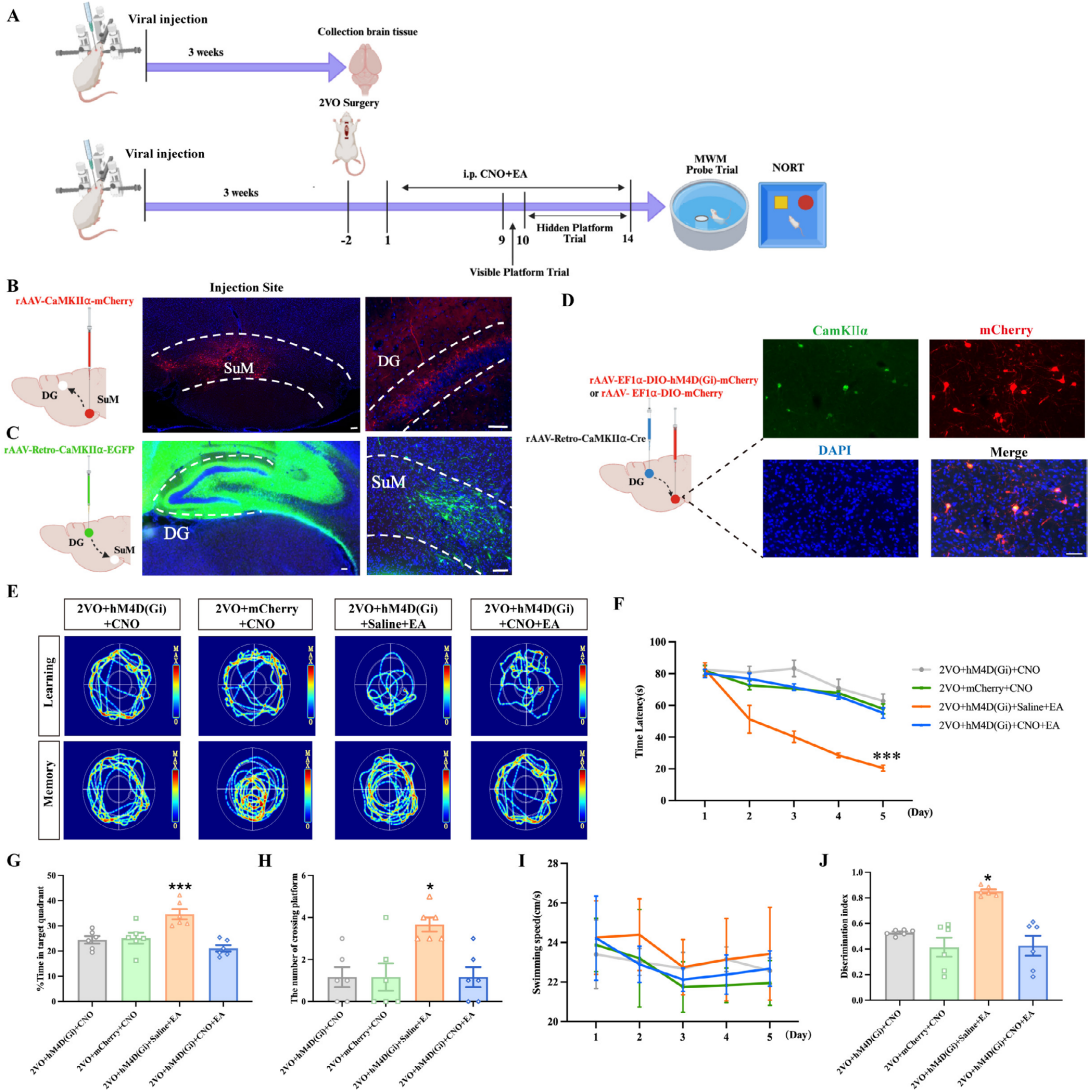
Chemogenetic inhibition of the SuM^Glu^‐DG circuit abolished the EA‐induced recovery. (A) Timeline of the experimental procedure. (B) Methodological representation of anterograde viral tracing technique (left) and typical images showing SuM inoculation sites and DG fluorescence signals (right). Scale bar: 200 μm. (C) Methodological representation of retrograde viral tracing technique (left) and typical images of confirming DG viral placement and somatic SuM expression (right). Scale bar: 200 μm. (D) Procedural diagram for chemogenetic viral administration (left) and representative images of mCherry co‐labeled with CAMKIIα expression in the SuM (right). Scale bar: 50 μm. (E) Representative swimming trajectories during the MWM test. (F) The escape latency to find the hidden platform in the place navigation phase (learning) (Two‐way ANOVA, *n* = 6 rats per group, Time: F_4,80_ = 43.685, *P* < 0.001; Groups: F_3,20_ = 63.678, *P* < 0.001; Group*Time: F_12,80_ = 5.409, *P > 0.05*; Day 5: One‐way ANOVA, *n* = 6 rats per group, F_3,20_ = 14.089, *P < 0.001*, post hoc Dunnett's test: ***P < 0.001 vs. 2VO + hM4Di + CNO + EA group). (G) Percentage of swimming time in the target quadrant during the probe test (memory) (One‐way ANOVA, *n* = 6 rats per group, F_3,20_ = 10.518, *P < 0.001*, post hoc Tukey's test: ***P < 0.001 vs. 2VO + hM4Di + CNO + EA group). (H) The number of platform crossing in the probe test (memory) (One‐way ANOVA, *n* = 6 rats per group, F_3,20_ = 6.285, *P < 0.01*, post hoc Tukey's test: *P < 0.05 vs. 2VO + hM4Di + CNO + EA group). (I) Average swimming speed in the MWM test (Two‐way ANOVA, *n* = 6 rats per group, Time: F_4,80_ = 0.999, *P > 0.05*; Groups: F_3,20_ = 0.269, *P > 0.05*.; Group*Time: F_12,80_ = 0.092, *P > 0.05*; Day 5: One‐way ANOVA, *n* = 6 rats per group, F_3,20_ = 0.269, *P > 0.05*). (J) The discrimination index in the NORT (One‐way ANOVA, *n* = 6 rats per group, F_3,20_ = 14.089, *P < 0.001*, post hoc Dunnett's test: *P *< 0.05* vs. 2VO + hM4Di + CNO + EA group).

To explore the connection between SuM^Glu^ and DG, anterograde tracing via rAAV‐CaMKIIα‐mCherry injection in the SuM revealed an abundance of mCherry + fibers within the DG (Figure 4B), indicating that SuM^Glu^ projects to DG. Conversely, retrograde tracing using rAAV‐Retro‐CaMKIIα‐EGFP in the DG identified a robust population of EGFP + neurons in the SuM (Figure 4C). These reciprocal tracing results confirm a direct glutamatergic projection from the SuM to the DG, providing the structural substrate for functional modulation.

To determine whether the SuM^Glu^‐DG circuit mediated the therapeutic effects of EA, the chemogenetic manipulation was performed (Figure 4A). The rAAV‐Retro‐CaMKIIα‐Cre virus was injected into the DG, and rAAV‐EF1α‐DIO‐hM4D(Gi)‐mCherry or rAAV‐EF1α‐DIO‐mCherry (serving as the control) was injected into the SuM. Results confirmed that the mCherry^+^ neurons were co‐localized with the CAMKIIα neurons (Figure 4D). The SuM^Glu^‐DG circuit was specifically inhibited after CNO administration. In the MWM test, two‐way ANOVA revealed significant differences of the escape latency in time and groups, but no interaction was detected in time × groups. As shown in Figure 4E–H, while EA treatment significantly improved spatial learning and memory in VCI rats, this benefit was nearly abolished upon chemogenetic inhibition of the SuM^Glu^‐DG circuit. Rats in the 2VO + hM4Di + CNO + EA group displayed significantly increased escape latencies, reduced target quadrant occupancy, and fewer platform crossings compared to the 2VO + hM4Di + Saline + EA group. No significant difference was observed between the 2VO + mCherry + CNO and 2VO + hM4Di + CNO groups, indicating that inhibition of the SuM^Glu^‐DG circuit did not further exacerbate memory impairment. No differences in average swimming speed were detected among the four indicated experimental groups in the MWM test (Figure 4I). The NORT (Figure 4J) demonstrated that the 2VO + hM4Di + CNO + EA group exhibited a lower discrimination index compared to the 2VO + hM4Di + Saline + EA group, indicating impaired non‐spatial memory. The 2VO + mCherry + CNO group presented a decreased discrimination index similar to the 2VO + hM4Di + CNO group, suggesting that inhibition of SuM^Glu^‐DG circuit did not further impair non‐spatial memory. These findings identified the SuM^Glu^‐DG circuit as an indispensable neural pathway for the therapeutic benefits of EA in VCI.

## 4 Discussion

The present study demonstrated that EA effectively mitigated learning and memory deficits in VCI rats by recruiting a specific hypothalamic‐hippocampal circuit. Specifically, we found that EA restored both synaptic plasticity and neuronal activity within the DG, providing a structural and functional basis for cognitive recovery. Beyond localized hippocampal repair, we identified the activation of glutamatergic neurons in the SuM as a critical upstream driver of these therapeutic benefits. Importantly, the fact that chemogenetic silencing of the SuM^Glu^‐DG circuit abolished the behavioral improvements induced by EA confirms that this circuit is indispensable for EA‐mediated neuroprotection.

The high degree of synaptic plasticity in the hippocampus supports cognitive functions while increasing vulnerability to pathological conditions [25]. During cerebral ischemia and hypoxia, neuronal activity and dendritic morphology are impaired. This impairment diminishes the capacity of the hippocampus to adapt and maintain cognitive functions, ultimately contributing to cognitive decline. The DG plays a crucial role in functions essential for effective learning and memory. We found that EA significantly improved impaired synaptic plasticity of the DG in rats following 2VO, resulting in amelioration of their learning and memory. Previous studies have demonstrated that EA restores the neuronal activity in the hippocampus, thereby ameliorating cognitive decline [26, 27]. Therefore, we further investigated whether the DG neuronal activity mediates the effects of EA. In vivo experiments were performed using fiber photometry to directly record DG neuronal activity in real time. We found that EA significantly enhanced neuronal activity in the DG region. Distinct from prior research, we conducted recordings at multiple time points (days 1, 7, and 14) throughout the EA intervention period. We found that the modulatory effect of EA on neuronal activity gradually intensified as the duration of EA treatment prolonged, indicating a time‐dependent enhancement of EA's neuroregulatory efficacy. Mechanistically, our real‐time functional dynamics and endpoint structural findings reflect an integrated process of activity‐dependent structural plasticity. The progressive elevation of calcium signal across the 14 day EA intervention likely serves as the functional substrate for the morphological rescue of dendritic spines. This structure–function coupling ultimately stabilizes the neuroprotective circuitry, underpinning the sustained cognitive recovery in VCI rats.

The SuM, a vital hypothalamic subregion, has emerged as a key player in high‐order cognitive processes, particularly through its rhythmic modulation of hippocampal activity [28]. A recent investigation revealed that SuM hypoactivity is closely linked to deficits in alertness and cognitive performance [29]. In the VCI model, we observed a significant suppression of SuM neuronal activity, which likely reflects the widespread impact of chronic hypoperfusion on subcortical regulatory centers. Therefore, we subsequently focused on whether the SuM mediates the therapeutic efficacy of EA. By utilizing c‐Fos mapping and fiber photometry, we confirmed that EA specifically activated SuM^Glu^ neurons, which suggested that the neuroprotective influence of EA is not merely the hippocampal phenomenon but involves the recruitment of upstream regulatory hubs. Accordingly, we speculated that the SuM^Glu^‐DG circuit may be critical for the neuroprotective effects of EA. Anatomical studies have demonstrated dense connectivity between glutamatergic neurons in the SuM and the DG [30, 31], which is consistent with our results. To corroborate the functional significance of the SuM^Glu^‐DG circuit, we inhibited this circuit in VCI rats using chemogenetic manipulation. Our data revealed that attenuating the SuM^Glu^‐DG circuit abolished the neuroprotective benefits of EA, establishing this circuit as a vital conduit for acupuncture‐mediated recovery.

A notable feature of the DG is its functional segregation along the dorsoventral axis: the dorsal DG (dDG) is primarily dedicated to spatial and contextual memory, whereas the ventral DG (vDG) is fundamentally implicated in the regulation of emotional states, notably encompassing anxiety and depressive‐like manifestations [32, 33, 34]. Recent research [35] has identified segregated SuM neuronal populations that target the dDG and vDG to selectively gate cognitive and affective functions. Furthermore, the SuM provides a ‘novelty signal’ to the DG that is indispensable for the encoding of new environmental information [12, 35]. In the present study, EA treatment specifically activated SuM glutamatergic neurons and significantly improved memory—such as the discrimination of novel objects in the NORT. Combined with the unchanged anxiety‐like behaviors or locomotor activity in the OFT, we propose that the therapeutic effects of EA are primarily mediated through the SuM‐dDG circuit, potentially by restoring these novelty‐related signals to facilitate memory encoding. While we did not perform subregion‐specific manipulations in the current work, our behavioral results align closely with the known role of the SuM‐dDG circuit in processing novelty and memory consolidation. Future studies employing targeted dDG and vDG interventions will be essential to directly validate this circuit‐specific hypothesis.

Pathological neuronal hyperexcitability, defined by an overabundance of spontaneous or evoked action potentials, substantially accelerates neurodegenerative processes and cognitive decline [36, 37]. Our results showed that EA enhanced neuronal activity in the DG, but the specific types of activated neurons have not been characterized to date. Previous studies have shown that SuM neurons innervate the DG by coreleasing two opposing neurotransmitters, glutamate and GABA, thereby supporting spatial navigation and memory [38]. However, the release ratios of these two neurotransmitters differ significantly depending on the subtype of DG target cells. The DG comprises excitatory granule cells (GCs) as well as inhibitory interneurons (INs). It is noteworthy that dendrite‐targeting INs (D‐INs) are the primary activation targets of the SuM glutamatergic neurons [39]. Upon activation, D‐INs modulate GCs via dendritic inhibition, which collectively enhances long‐term potentiation induction. In addition, glutamate (but not GABA) release from SuM neurons plays a critical role in regulating spatial memory retrieval [40]. Based on these findings, we suppose that EA exerts its therapeutic effects by activating SuM glutamatergic neurons, which in turn mediate the activation of D‐INs (GABAergic interneurons). This hypothesis aligns with our results that EA increased neuronal activity in SuM and DG, and improved synaptic plasticity, though direct validation was lacking in the current study. Therefore, we intend to undertake more rigorous investigations to elucidate this potential mechanism in future studies.

Despite the promising findings, several limitations of the present study should be acknowledged. First, while our endpoint data indicate that spontaneous recovery in untreated 2VO rats is highly unlikely, the lack of a longitudinal 2VO control in our fiber photometry recordings prevents us from completely excluding subtle spontaneous hemodynamic fluctuations. Second, systemic CNO administration inhibits SuM somata, meaning we cannot entirely rule out parallel functional effects from axon collaterals projecting to other regions (e.g., CA2/CA3). Future studies incorporating persistent non‐intervention controls and local CNO microinfusion into the DG will be valuable to further refine these circuit‐specific observations.

In summary, we found that EA intervention restored impaired synaptic plasticity and improved cognitive function in VCI rats, a therapeutic effect mediated by activation of the SuM^Glu^‐DG circuit. The present research offers new perspectives into the mechanism underlying the clinical efficacy of acupuncture in neuroprotection.

## Author Contributions

Fang Wan: writing – original draft, visualization, validation, formal analysis. Lu Wang: writing – review and editing, methodology, validation, funding acquisition. Qing‐Yong Wang: methodology, investigation, formal analysis. Jing‐Wen Yang: supervision, conceptualization, funding acquisition. Cun‐Zhi Liu: writing – review and editing, supervision, conceptualization.

## Funding

This work was supported by the National Natural Science Foundation of China [Grant number 82004479] and the China Postdoctoral Innovation Talent Support program [Grant number BX20250215] and the China Postdoctoral Science Foundation [Grant number 2025M783954].

## Ethics Statement

All experiment were approved by the Animal Care and Use Committee of Beijing University of Chinese Medicine (Permission Number: BUCM‐4‐2020091601‐3063).

## Conflicts of Interest

The authors declare no conflicts of interest.

## Data Availability

The data that support the findings of this study are available on request from the corresponding author. The data are not publicly available due to privacy or ethical restrictions.
